# Comparing the Diagnostic Accuracy of Simple Tests to Screen for Diabetic Peripheral Neuropathy: Protocol for a Cross-Sectional Study

**DOI:** 10.2196/resprot.7438

**Published:** 2018-04-06

**Authors:** Kate Goddard, Prashanth Vas, Alistair Purves, Viktoria McMillan, Thomas Langford, Fiona Reid, Michael Edmonds

**Affiliations:** ^1^ King's Technology Evaluation Centre King's College London London United Kingdom; ^2^ King's College London Hospital NHS Trust London United Kingdom

**Keywords:** peripheral nervous system diseases, diabetes mellitus, diabetic foot, diabetic neuropathies, predictive value of tests, sensory thresholds, sensitivity and specificity

## Abstract

**Background:**

Various tests are used to detect diabetic peripheral neuropathy by assessing sense perception in the feet. Tests vary in terms of time and resources required. Simple tests are those that can be conducted quickly and easily in primary care without laboratory equipment. There are some limitations to these simple tests, an example being the variable amplitude of the 128 Hz tuning fork. A new test, VibraTip (McCallan Medical, UK), might be a valuable alternative as it emits a consistent amplitude and may offer improved diagnostic accuracy.

**Objective:**

The aims of this study are to estimate the diagnostic accuracy of the VibraTip device for diabetic peripheral neuropathy against the reference standard of sural nerve conduction velocity measurement, and to assess whether the VibraTip offers superior diagnostic accuracy to other routine tests based on vibration or touch.

**Methods:**

The study will prospectively recruit adults with type 2 diabetes who are due to attend a routine follow-up clinic. A cross-sectional study design will be employed to assess the diagnostic accuracy of 5 standard index tests for peripheral neuropathy, including VibraTip. The reference test will be sural nerve conduction velocity measurement.

**Results:**

Funding is being sought to conduct this research. The outcomes assessed will be the diagnostic accuracy of the 5 index tests against sural nerve conduction velocity measurement, including sensitivity, specificity, positive predictive value, negative predictive value, positive likelihood ratio, and negative likelihood ratio. Receiver operating characteristic curves will be constructed and compared for each test.

**Conclusions:**

This study will be the first within-study comparison of 5 simple tests for screening diabetic peripheral neuropathy and will address uncertainties in the potential benefits of using VibraTip in comparison with the other tests.

## Introduction

### Background

Diabetic peripheral neuropathy (DPN), nerve damage caused by poorly controlled high blood sugar levels, is the most common complication of diabetes, affecting as many as 50% of people with the disease [1]. DPN can lead to loss of protective sensation in the feet, which is associated with an increased risk of ulceration. Diabetic foot ulcers can become infected and gangrenous and this, ultimately, leads to major (above or below the knee) or minor (toe or foot) lower limb amputations; indeed, a nonhealing ulcer precedes 85% of such amputations [2]. Diabetes UK has calculated that the number of diabetes-related amputations in England has now reached an all-time record high of 140 per week, equating to approximately 7400 per year [3].

The National Institute for Health and Care Excellence (NICE) Medical Technologies Guidance (MTG) 22 [4] notes that detection, diagnosis, and management of DPN is an important clinical area which has the potential to affect millions of people in the UK. Specifically, foot ulcers cause substantial emotional, physical, and financial losses. Total National Health Service (NHS) spending on ulceration in people with diabetes in England in 2014–15 was estimated at £650 million, equivalent to 0.6%–0.7% of NHS expenditure [5]. Every individual amputation costs the NHS between £8011 and £16,136, depending on whether the patient has comorbidities or develops complications [6].

The prevention of foot ulceration and amputation serves as an effective cost-saving strategy by avoiding the need for expensive interventions such as treatment of foot ulcers and infections, leg amputations, and lower extremity revascularisation procedures [7]. Moreover, beyond the direct costs to the health care sector, there are a number of costs directly incurred by individuals, employers and society in general such as costs for drugs, those indirectly incurred costs due to lost productivity and personal /carer distress. Therefore, even small improvements in the timing and characterisation of DPN detection may have the potential for substantial impact.

Prospective studies have demonstrated that screening for DPN can successfully predict people at risk of ulceration [8]. Vas et al [9] provide a review of the current techniques that may be used for diagnosing DPN. Various simple tests are used to detect DPN by assessing sense perception (either vibration or touch) in the feet. More complex tests, with nerve conduction studies (NCS) only accessible in neurophysiology laboratories, are typically used as the gold standard for diagnosis (noting, however, that there is no universally agreed gold standard). There are often restrictions in time and resource for carrying out in-depth screening in primary care settings where expertise and equipment is unavailable and in busy diabetic clinics when time is constrained and the focus is on fast, simple methods and technologies. The more complex methods tend to be used when clinical presentation is atypical.

In the UK, there is no standardized method of assessing DPN; however, it is typically assessed by a touch test using simpler methods such as 10 gram monofilament, or a vibration test using a 128 Hz tuning fork (NICE MTG 22). The widely-used monofilament [10] has limitations. For example, it needs to be rested for 24 hours after 10 applications and replaced after 100 applications. Furthermore, not all monofilaments available commercially apply 10 g of force and enforcing quality control has been difficult. The tuning fork has one major disadvantage; it constantly decreases in amplitude during its application. Other common tests involve using a neurothesiometer (vibration), which is expensive and not widely used in primary care, or the Ipswich Touch Test (IpTT; touch), which has been validated only in a hospital setting for foot risk prediction. Newer, simple techniques are being developed to help accurately screen for DPN outside of the neurophysiology clinic. One such device, the VibraTip (McCallan Medical, UK; Conformité Européene [CE] marked in 2010) resembles a small keyring fob that provides a near-silent vibration at a frequency similar to that of a calibrated tuning fork, but with a consistent amplitude.

The VibraTip device has been the subject of previous NICE guidance reports. NICE MTG 22 states that VibraTip shows potential to improve the detection of DPN and to provide cost savings to the NHS. The guidance states that VibraTip appears to be easy to use, portable, and reliable in its functionality, but that current evidence is insufficient to support the case for its routine adoption by the NHS. Population size in the studies outlined in the literature review in NICE MTG22 varied between 42 and 496 participants. Studies compared 2-5 index tests against 0-2 reference standards. Bracewell et al [11] was deemed the best quality study in the MTG22 summary of the clinical evidence. MTG22 questioned whether the sample size was adequate (n=141) to assess 4 index tests against 1 reference standard (neurothesiometer). The MTG22 guidance suggests that previous studies [11-16] were of insufficient methodological quality to provide conclusive evidence (eg, sample sizes tended to be small, and inappropriate reference standards were used) and had a high risk of bias. Therefore, research is recommended to address uncertainties in the potential benefits of using VibraTip to patients and the NHS.

As far as the authors are aware, this proposed research would also provide the only within-study comparison of the accuracy of 5 simple and (relatively) commonly used tests for DPN. There are no other published, completed or ongoing studies that compare typically-used methods of assessing DPN with a reference standard of SNCV or neurophysiology, and also address potential biases in previous studies.

### Objectives

This study has two aims: (1) to estimate the diagnostic accuracy of VibraTip in detecting DPN against the reference standard of SNCV measurement, and (2) to assess whether the VibraTip device offers superior accuracy compared with 4 other routine tests for peripheral neuropathy (see Table 1 for a description of the tests; these include both touch and vibration tests).

**Table 1:** Index and reference test schedule. SNCV: sural nerve conduction velocity.

Test	Test type	Is the test typically given to participants as part of care outside research?	Average time per test procedure	Who will conduct the test and where	Description of test procedure^a^
VibraTip	Vibration	Yes	5 min	Clinician; diabetic follow-up clinic	Ten sites on each foot will be tested. The VibraTip is applied to the patient’s foot twice: once while not vibrating and once while vibrating. The patient is asked to indicate when they feel vibration. The VibraTip should be applied for 1 second in each instance.
Monofilament (10 g)	Touch	Yes	5 min	Clinician; diabetic follow-up clinic	Ten sites on each food will be tested. The monofilament is lightly pressed to the skin so that it buckles into a C-shape. The patient is asked to indicate whether they feel the touch. The monofilament should be applied for 1 second in each instance.
Tuning fork (128 Hz)	Vibration	Yes	5 min	Clinician; diabetic follow-up clinic	Per typical practice, 3 sites will be tested: tip of hallux on each foot, medial malleolus on each ankle, and each knee (6 sites in total). The 128 Hz tuning fork is struck before being applied to the feet at each site for 1 second.
Neurothesiometer set at ≥25 V	Vibration	Yes	10 min	Clinical scientist; diabetic follow-up clinic	The neurothesiometer will be set at 25 V (vibration perception threshold, VPT) and failure to detect vibration at this VPT indicates neuropathy. In Bracewell et al [11], detection of ≥25 V using the neurothesiometer was assessed at the pulp of the hallux (great toe) only. This will be assessed on each foot.
Ipswich Touch Test (IpTT)	Touch	Yes	5 min	Clinician; diabetic follow-up clinic	The IpTT involves very lightly touching 6 toes, 3 on each foot to find out how many of the touches are felt. Touch will last for 1 second. Each touch will not be repeated (ie, no toes much be touched more than once). Normal sensation is indicated if touch was felt in at least 5 of 6 toes; fewer than this indicates neuropathy.
SNCV measurement (reference test)	SNCV	No	5-15 min	Clinician or clinical technician; neurology department	This involves 2 electrodes being applied to the patient’s skin: one at the knee and one at the ankle. The first electrode sends a small painless electrical impulse through the nerve. The second electrode records the impulse. The time difference between the impulse being sent by the first electrode and being received by the second electrode indicates how quickly the sural nerve is transmitting electrical impulses. If the speed at which the impulse is transmitted is abnormal, this is an indication of diabetic peripheral neuropathy. Additionally, bilateral sural nerve amplitude and superficial peroneal amplitude measurements may also be considered within the same session to increase the accuracy of the reference standard. Skin temperature will be kept at a standard level, verified by a skin thermometer, and measurement will be bilateral.

^a^Tests are described in detail by Papanas and Ziegler (2014) [17].

## Methods

### Type of Study

The study population will be recruited prospectively. A cross-sectional study design will be used to assess the diagnostic accuracy of 5 simple methods of identifying peripheral neuropathy against SNCV measurement as reference standard. This study design will provide a like-for-like comparison of the tests within the same participants. Ideally, patients will only be participating in the study for one day at the time of their routine follow-up appointment.

### Setting

For a UK study, settings should include centers that carry out routine screening for diabetic complications. This may be at a general practitioner clinic or, more typically, a diabetes clinic. The researchers will require access to a neurology department within which NCS testing can be carried out by qualified staff. Ideally, this will be within the same center to minimize the time between the carrying out of standard tests and the SNCV assessment.

### Inclusion and Exclusion Criteria

The only inclusion criterion is that study participants must be adults with type 2 diabetes mellitus who have no history of foot ulcers.

The following exclusion criteria will be used:

- Patients who already have ulcerations (or a history of ulcers) or amputations
- Patients under 18 years of age
- Those unable to provide consent or a satisfactory response because of cognitive impairment (eg, people with dementia are considered high risk)

### Study Procedure

Diabetic patients, who are due to attend routine follow-up clinic, will be screened for eligibility according to the inclusion and exclusion criteria outlined above. Screening may be undertaken by an investigator with permission to access the organization’s patient administration system for the diabetes service. Patients will be aware of test results during the testing procedures, but will be asked to close their eyes while the procedures are carried out.

**Figure 1:**
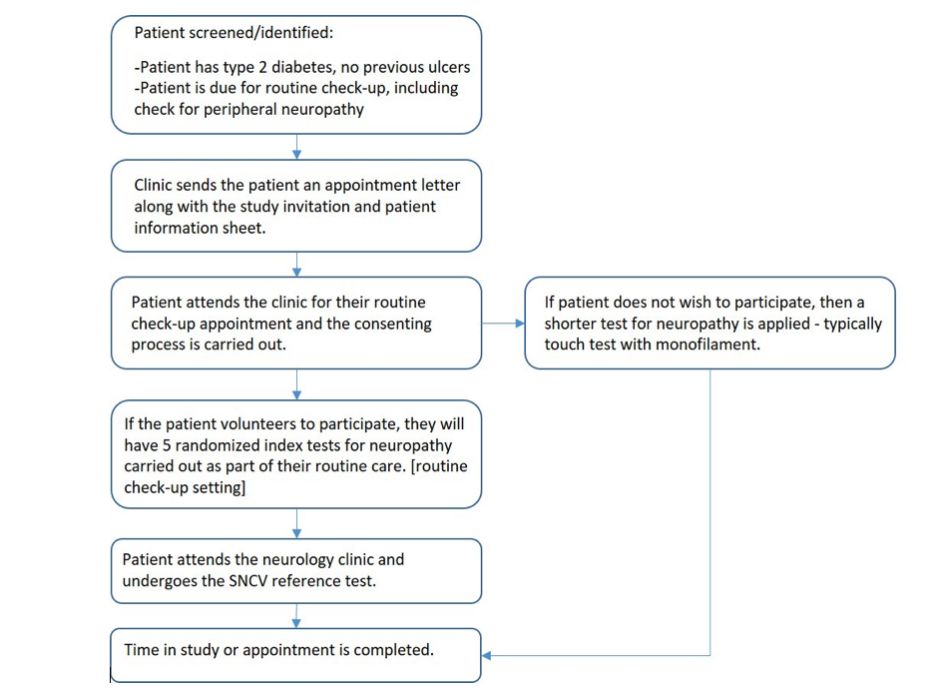
Study process per patient. SNCV: sural nerve conduction velocity.

Due to the comparative complexity of setting up the SNCV measurements, this will occur independently of the index tests. Patients will arrive at the neurology department to undergo SNCV measurement, which will take approximately 5-15 min. This may be carried out on the same day (ideally, if the neurology department is close to or within the recruiting clinic), or on a different day if this is not possible. To minimize interexaminer variation, the nerve tests should ideally be carried out by the same examiner, or by a controlled number of neurology staff working based on agreed protocols. The process for the patient is illustrated by Figure 1.

### Outcome Measures

For the vibration- and touch-based tests, the outcome measure will be the number of sensate and the number of insensate sites per patient. For the SNCV test, the outcome measure will be the speed of sural nerve conduction in meters per second.

### Number of Test Sites

There is no agreed standard on the number or location of sites on each foot that should be examined [4]; published literature describes different approaches varying from 1 testing site to 10 testing sites per foot [1]. Bracewell et al [11] assessed the optimal number of sites on the feet that should be tested to detect peripheral neuropathy using VibraTip, 10 g monofilament and 128 Hz tuning fork by testing 5 sites on each foot. Their analysis suggested that finding ≥2 insensate sites across the 10 sites (5 on each foot) may be indicative of peripheral neuropathy for VibraTip and monofilament, whereas ≥1 insensate sites across 10 sites in 2 feet may be indicative of peripheral neuropathy for the 128 Hz tuning fork.

To perform tests thoroughly and be able to assess the accuracy of testing at different thresholds for the VibraTip and monofilament, this study will test 10 sites per foot (20 overall per participant). The 128 Hz tuning fork will be tested in 6 sites as per typical practice. The IpTT and neurothesiometer also have more standardized procedures (testing 6 and 2 sites across 2 feet, respectively), which will be followed for this study. The data from this will be used to perform receiver operating characteristic (ROC) analyses for each of the vibration and touch tests compared with SNCV, to assess the optimum number of insensate sites that give the best diagnostic accuracy.

### Test Randomization

Repeated touch of the same area on the patient’s foot may affect sensitivity to subsequent tests. As a mitigating measure, breaks of at least 5 minutes will occur between each test. The order of the index tests will also be randomized.

### Data Collection

A specific proforma has been created to collect data for this study (see Multimedia Appendix 1). This proforma will be used in parallel with the routine case notes for the follow-up appointment. The clinician carrying out the routine follow-up will also be responsible for completing the proforma.

Each proforma (1 per participant) will have 7 pages with the following diagrams/guidance and space to record results:

1. Cover sheet: unique study number, order of tests (prerandomized), record of age, gender, and visit date
2. Index test A for VibraTip: simple illustration of 2 feet with areas marked for 10 sites to be tested on each foot
3. Index test B for 10 g monofilament: simple illustration of 2 feet with areas marked for 10 sites to be tested on each foot
4. Index test C for 128 Hz tuning fork: simple illustration of 2 feet, 2 ankles and 2 knees with areas marked for 6 sites per patient to be tested
5. Index test D for neurothesiometer: simple illustration of 2 feet with areas marked for 1 site to be tested on each toe
6. Index test E for IpTT: simple illustration of 2 feet with areas marked for 3 toe sites to be tested on each foot
7. Space for recording the result of the SNCV test

### Sample Size

Peripheral neuropathy can affect up to 50% of the population with type 2 diabetes [18]. The reported 0.79 sensitivity and 0.82 specificity from Bracewell et al [11] can be used as the estimated sensitivity and specificity for VibraTip. These are conservative estimates compared with those described by Bowling et al [12] and Nizar et al [15]. Using the Confidence Interval Analysis software package, a sample size of 102 and a sensitivity of 0.79 will give an acceptable 95% confidence lower limit for sensitivity of 0.7. Assuming half the population will be positive for neuropathy (true cases) and half will be negative for neuropathy, a total sample size of 204 patients will be required.

### Statistical Analysis

All analyses will be performed using the latest versions of SPSS, and the following outcomes will be assessed:

1. Primary outcomes: The sensitivity, specificity, positive and negative predictive values, and positive and negative likelihood ratios will be measured for each test, using established thresholds, and presented with 95% confidence intervals.
2. Secondary outcomes: The sensitivity and specificity of the five index tests will be compared using McNemar’s test for paired proportions. ROC curves will be constructed for each index test, using the full range of possible thresholds per test. Statistical significance of the difference between the areas under ROC curves (derived from the same cases) will be tested with the method of DeLong et al [19].

### Ethics and Governance

Patients due for a routine follow-up will be sent a patient information sheet and invitation to take part in the study along with their appointment letter. The date that the letter was sent will be recorded. The patient will receive this at least 48 hours before they attend clinic.

On attending their follow-up appointment, the patient will be asked whether they have read the invitation and patient information sheet. If they respond affirmatively and favorably to this, the investigator will explain the aims, methods, anticipated benefits, and any potential risks of the study. The patient will be able to ask any questions or highlight any concerns about the study. The investigator will explain to the potential participant that they are free to refuse any involvement in the study or withdraw their consent at any point during the study. Individual patient consent will be requested at entry by the recruiting clinician.

The study protocol has been given a favorable ethical opinion from an NHS Research Ethics Service by proportionate review.

## Results

Funding has been sought to carry out this proposed research. This study is expected to be completed in 2018.

## Discussion

### Study Rationale

This study aims to address the uncertainties identified in NICE MTG 22 by carrying out a prospective diagnostic accuracy study with a more robust reference standard than used in previous studies. There is no universally acknowledged reference method for diagnosing DPN, especially advanced DPN, which increases the risk of developing foot ulceration. The use of nerve NCS is typically viewed as the most acceptable option for confirming DPN. However, DPN is a length-dependent, axonal neuropathy, and therefore, assessment of the sural nerve (as proposed in this study)—the longest sensory nerve—may have the greatest face validity as a single parameter for its identification [18]. There is a paucity of studies comparing methods of diagnosis to the reference standard of NCS such as SNCV. This study should help provide a robust indication of the performance of commonly, and less commonly used methods of routinely testing for DPN.

Recommendations from NICE MTG22 prompted the development of this protocol. NICE may update its guidance if substantive evidence is generated on the superior accuracy of VibraTip in testing for DPN, thus addressing the evidence gaps identified in MTG 22. If the research outcome is favorable, updated NICE guidance would have a very strong influence on adoption nationally and internationally.

DPN is a serious condition that can lead to ulcers and amputation. These preventable outcomes are distressing to patients and costly to the health care system. Early identification and foot risk stratification will allow an increased window of opportunity to ensure at-risk patients are enrolled in an appropriate foot protection program. The evaluation of simple assessment methods may also provide more information for carrying out clinical and cost effectiveness analyses.

### Challenges

There are multiple potential methods of assessing DPN. The methods outlined in this protocol are, to the authors’ knowledge, the most typical of simple methods carried out in routine practice. This may not, however, be exhaustive. One device that has not been incorporated into this protocol, but may warrant consideration, is the handheld DPN-Check (Neurometrix, USA). The device is able to provide a point-of-care estimate SNCV and sural nerve conduction amplitude, and may be an acceptable proxy to standard NCS for screening and identification. The adoption of this device into routine clinical practice may, however, be limited by device complexity and precision [19].

Many other electrophysiological parameters exist and may be used in the assessment of DPN. However, though a combination of multiple tests may elicit an incrementally more accurate reference standard, research must also remain within the boundaries of practicality. For this reason, and to minimize the inconvenience to the patients who are attending clinic for routine assessment and will already be undergoing multiple index tests, SNCV is an appropriate choice. Additionally, bilateral sural nerve and superficial peroneal amplitude measurements may also be taken.

### Future Directions

The proposed study will be undertaken in an environment where the accuracy of different tests with the same patient can be assessed using more complex comparators as the reference standard compared with previous studies. These comparators require technical equipment and trained operators (not readily available in primary or community care). Should the VibraTip reliably demonstrate equivalent or superior accuracy to other index measures for DPN, the device may prove particularly useful in the primary or community care setting, and this may therefore be a key aim of further research.

Another potential factor for testing is the effect of the training and experience of the tester. Tests in the primary care and community settings may be performed by several different examiners (of unspecified levels of training and experience), and therefore, the issue of interrater variability should be investigated.

## Appendix

Multimedia Appendix 1 Data collection proforma.

## Abbreviations

CE: Conformité Européene
DPN: diabetic peripheral neuropathy
IpTT: Ipswich Touch Test
MTG: Medical Technologies Guidance
NCS: nerve conduction studies
NHS: National Health Service
NICE: National Institute for Health and Care Excellence
ROC: receiver operating curve
SNCV: sural nerve conduction velocity
VPT: vibration perception threshold
