# Supplementary material for: Comparing the Diagnostic Accuracy of Simple Tests to Screen for Diabetic Peripheral Neuropathy: Protocol for a Cross-Sectional Study
Source: JMIR Res Protoc. 2018 Apr 6;7(4):e72. doi: 10.2196/resprot.7438 (PMC5910530; doi:10.2196/resprot.7438)
Supplement: Multimedia Appendix 1 [file resprot_v7i4e72_app1.pdf]

[VERSION NUMBER]

[DATE]

VIBRATIP FOR THE DIAGNOSIS OF DIABETIC PERIPHERAL NEUROPATHY

DATA COLLECTION PROFORMA

Date of testing: ………/…………/……….

Participant identification number (e.g. 001): _ _ _

Age (years): …………

Gender: …………

ORDER OF TESTS:

1. VibraTip
2. 10g monofilament
3. 128Hz tuning fork
4. Neurothesiometer
5. Ipswich Touch Test
6. Sural nerve conduction velocity measurement [Neurology department]

KEY:

✓ = sensate

✗ = insensate

VIBRATIP

PLANAR

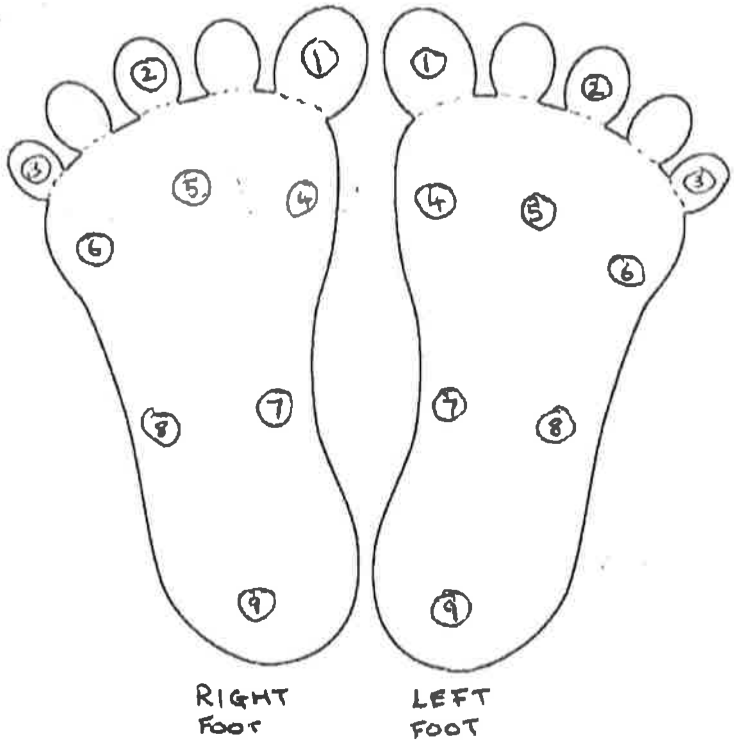

DORSUM

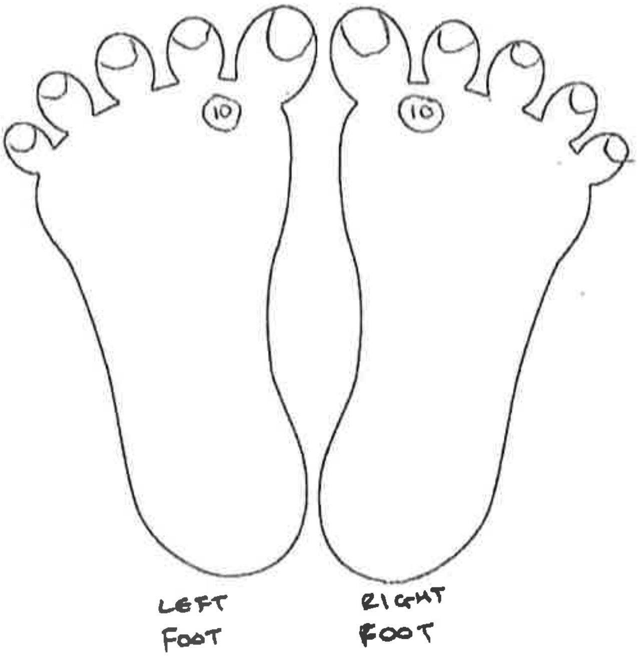

ADDITIONAL NOTES:

....

....

....

....

10G MONOFILAMENT

KEY:

✓ = sensate

✗ = Insensate

PLANAR

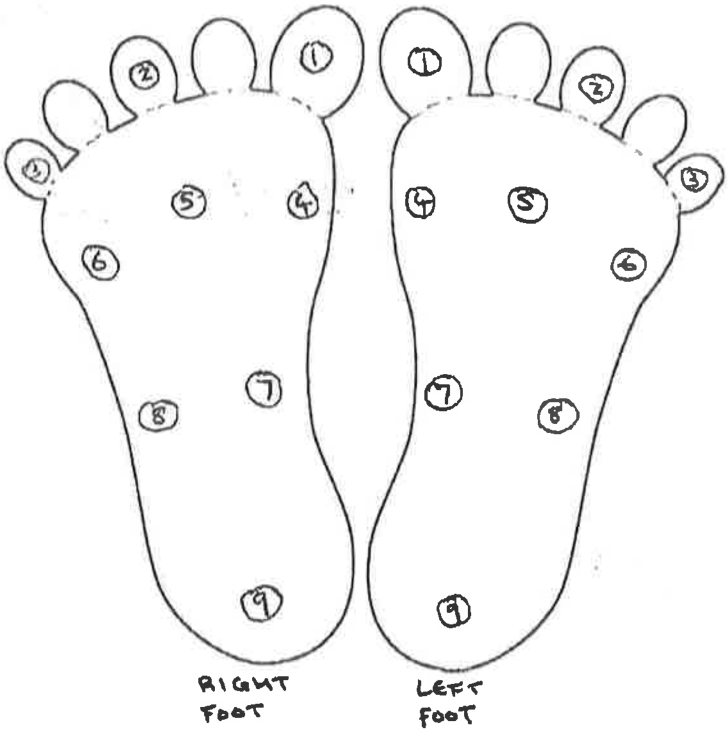

DORSUM

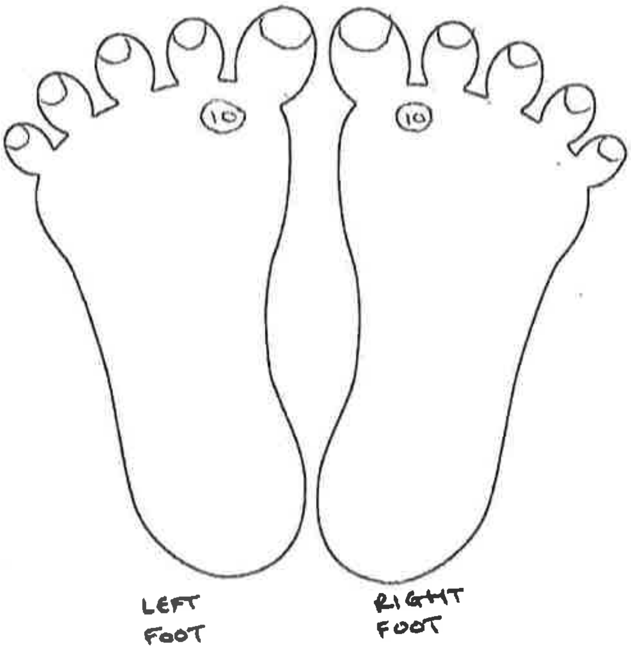

ADDITIONAL NOTES:

....

....

....

....

KEY:

✓ = sensate

✗ = Insensate

128Hz TUNING FORK

PLANAR

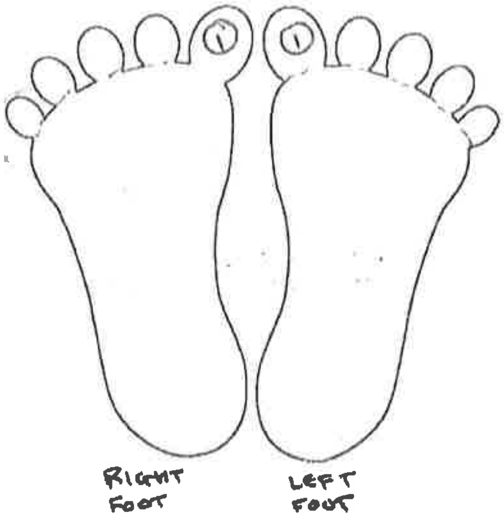

IF NO RESPONSE:

MEDIAL MALLEOLUS

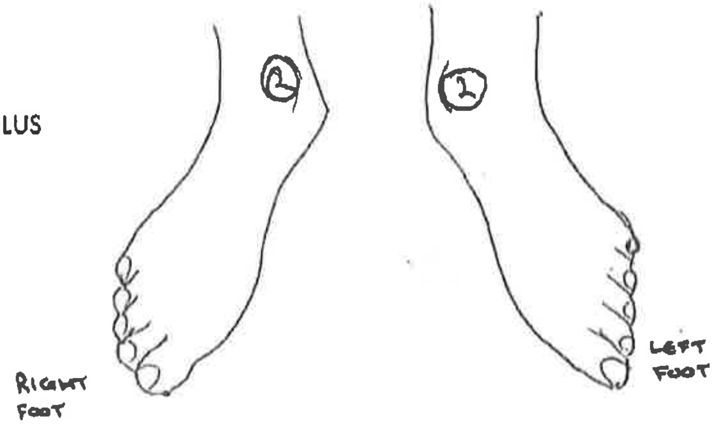

KNEE

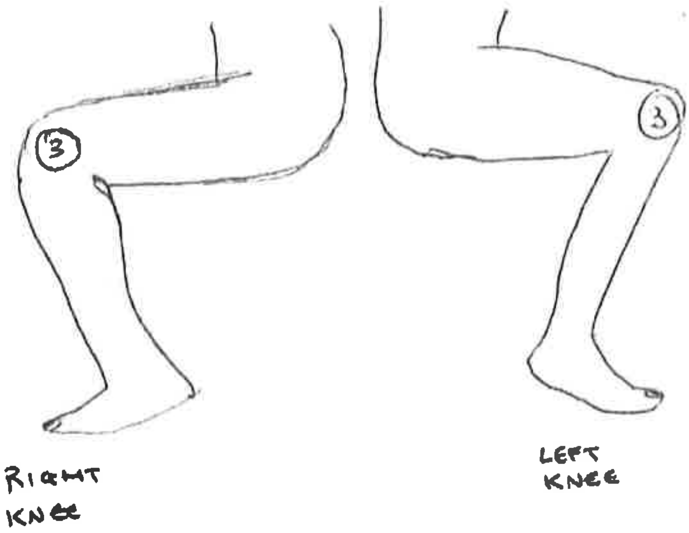

ADDITIONAL NOTES:

....

....

....

....

KEY:

✓ = sensate

✗ = Insensate

NEUROTHESIOMETER

PLANAR

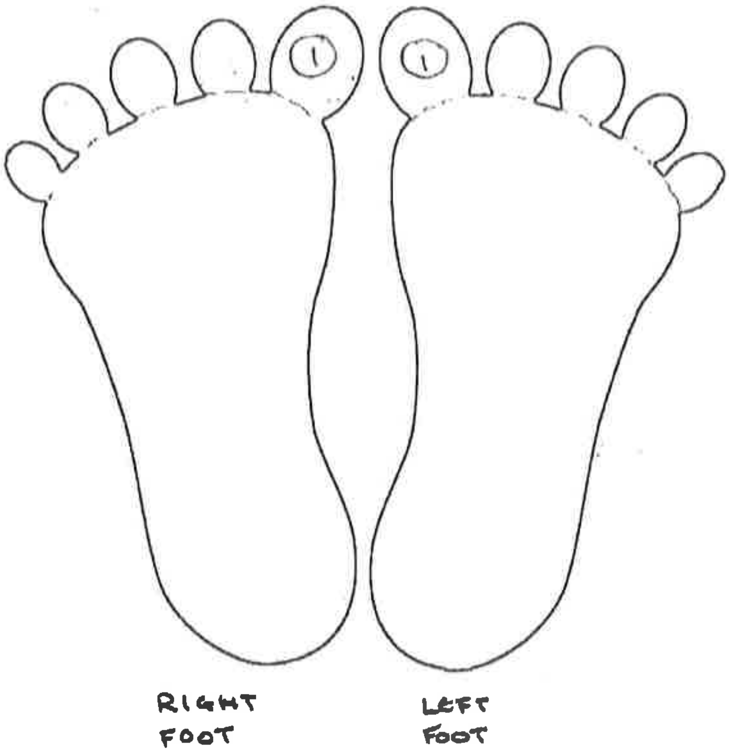

ADDITIONAL NOTES:

....

....

....

....

KEY:

✓ = sensate

✗ = Insensate

IPSWICH TOUCH TEST

PLANAR

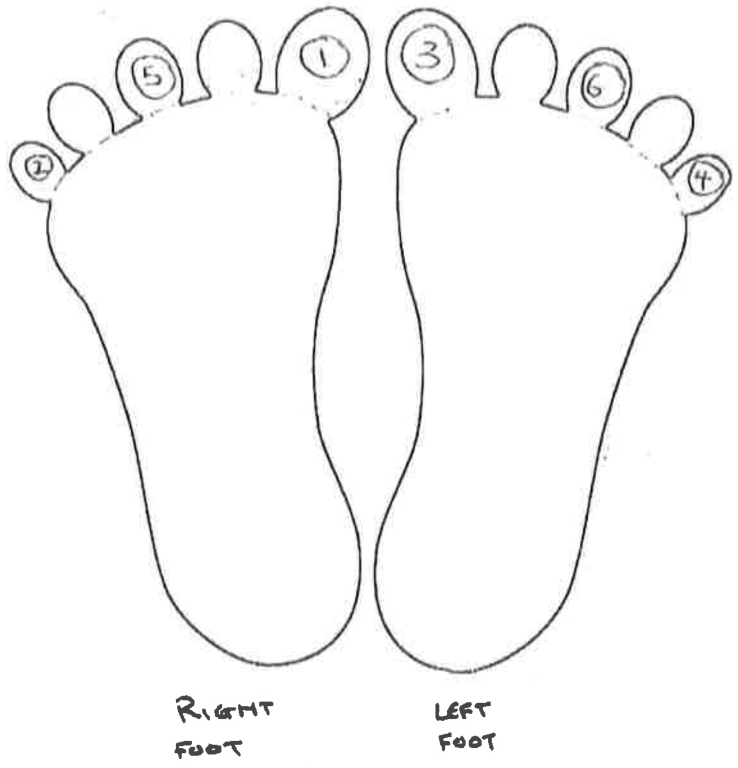

ADDITIONAL NOTES:

....

....

....

....

SNCV TEST

SNCV READING: _______ m/s

SNAP READING: _______ m/s

Abnormal: Y / N

ADDITIONAL NOTES:

....
....
....
....
